# Supplementary material for: Pharmacokinetics and Pharmacodynamics of Antibody-Drug Conjugates Administered via Subcutaneous and Intratumoral Routes
Source: Pharmaceutics. 2023 Apr 3;15(4):1132. doi: 10.3390/pharmaceutics15041132 (PMC10142912; doi:10.3390/pharmaceutics15041132)
Supplement: Supplementary file 1 [file pharmaceutics-15-01132-s001.zip › pharmaceutics-2209021-supplementary.pdf]

Type of the Paper (Article)

Pharmacokinetics and Pharmacodynamics of Antibody-drug Conjugates Administered via Subcutaneous and Intratumoral Routes

Supplementary Materials

Hsuan-Ping Chang [1], Huyen Khanh Le [1] and Dhaval K. Shah [1,*]

[1] Department of Pharmaceutical Sciences, School of Pharmacy and Pharmaceutical Sciences, The State University of New York at Buffalo, Buffalo, NY

* Correspondence: dshah4@buffalo.edu

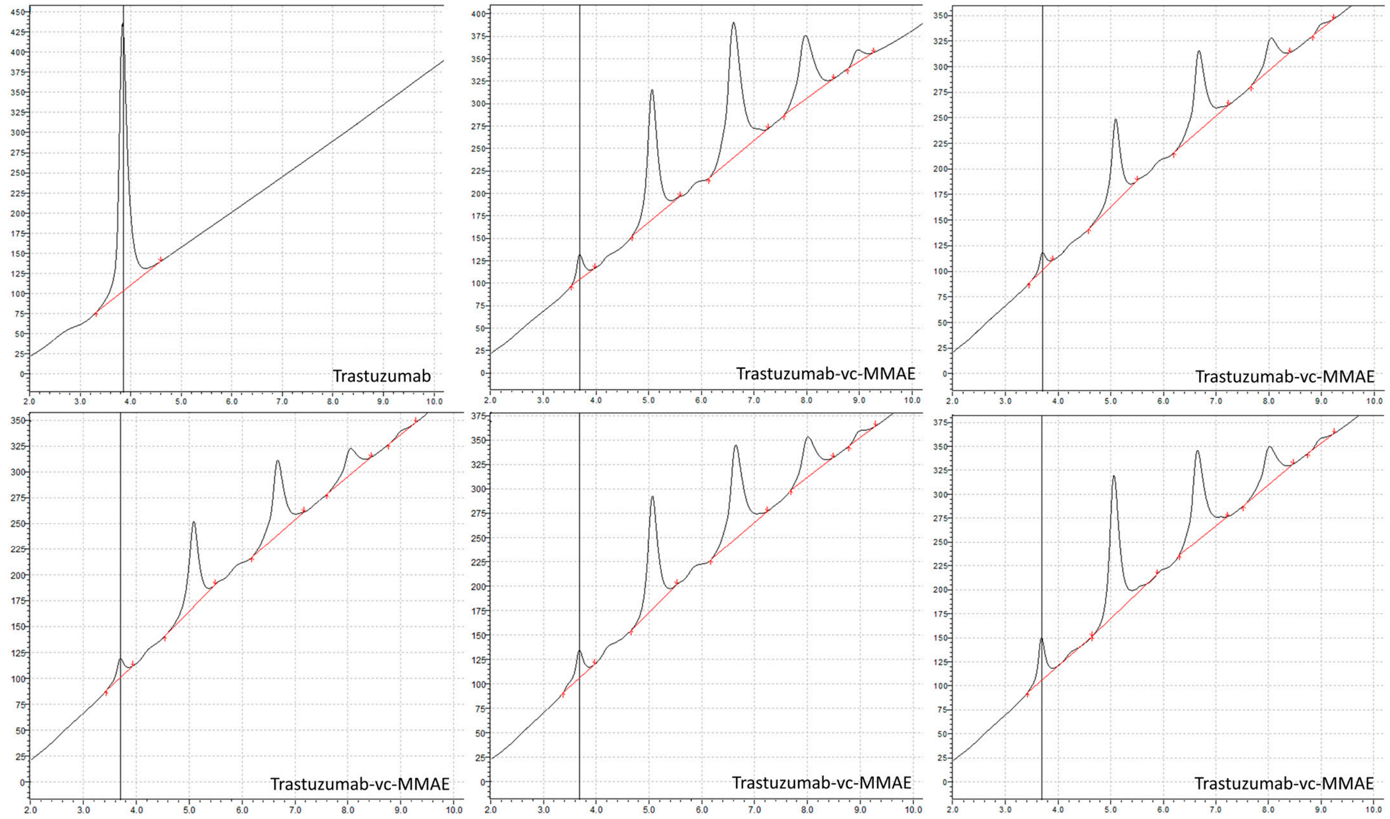

Figure S1. Hydrophobic interaction chromatography (HIC) analysis of T-vc-MMAE ADC for different batches used in this study. The average drug-antibody-ratio (DAR) from each batch was comparable. Trastuzumab as a control.

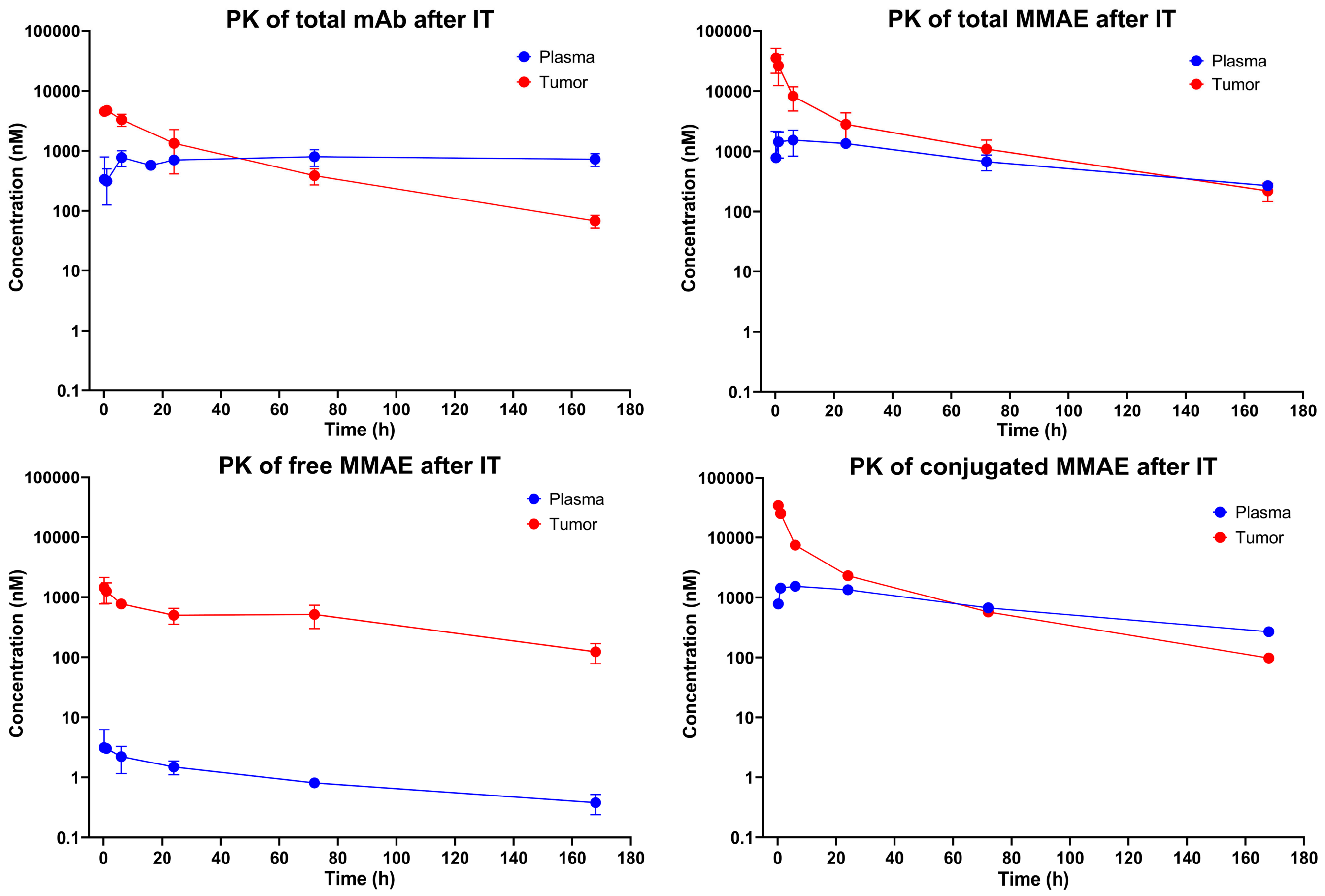

Figure S2. Observed plasma and tumor pharmacokinetics (PK) of ADC analytes in mice after intratumoral (IT) administration of 10 mg/kg of T-vc-MMAE single dose. The figure displays the mean (SD) observed concentration of: (**a**) Total antibody; (**b**) Total MMAE; (**c**) Unconjugated MMAE; (**d**) Conjugated MMAE in plasma and tumor.

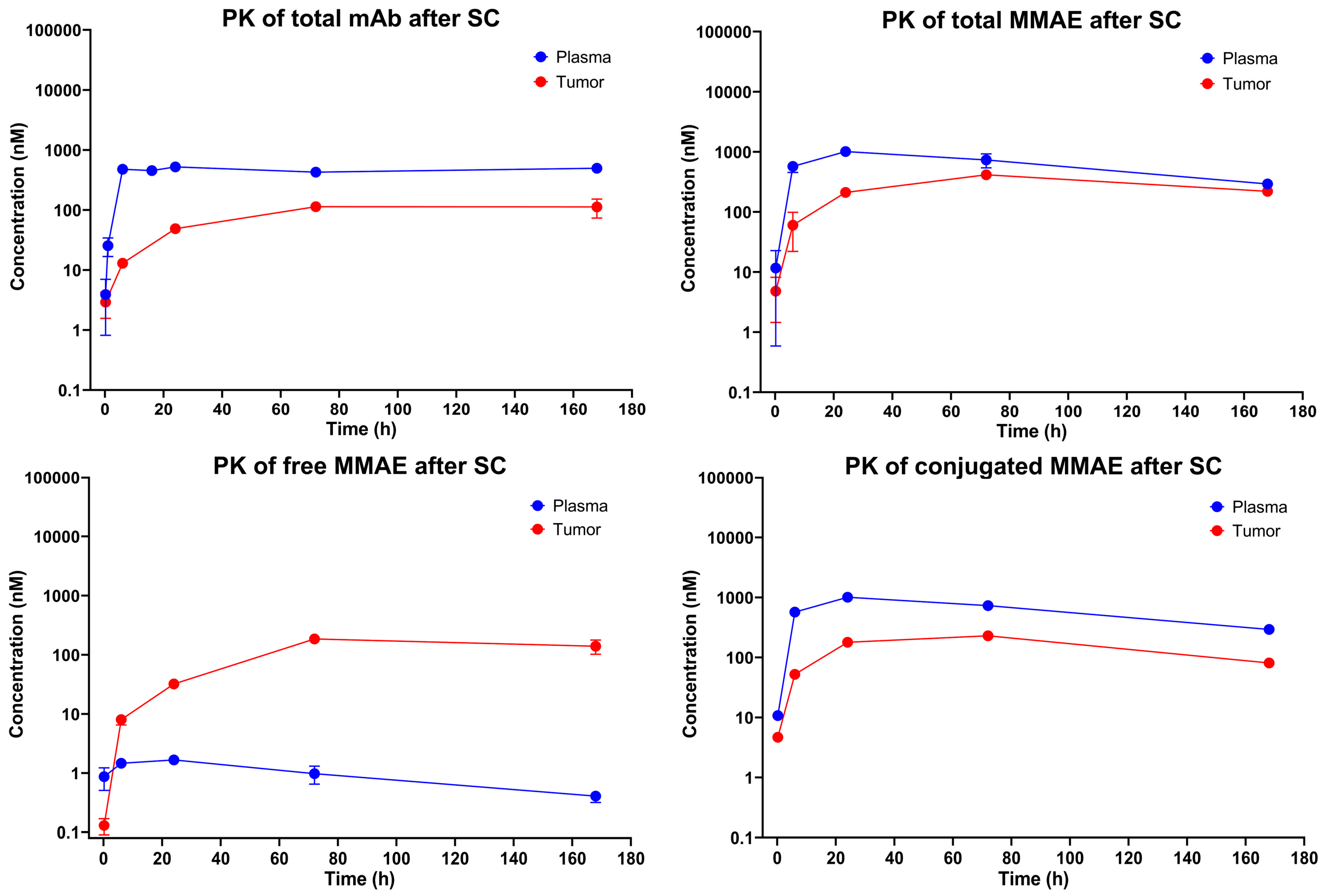

Figure S3. Observed plasma and tumor pharmacokinetics (PK) of ADC analytes in mice after subcutaneous (SC) administration of 10 mg/kg of T-vc-MMAE single dose. The figure displays the mean (SD) observed concentration of: (**a**) Total antibody; (**b**) Total MMAE; (**c**) Unconjugated MMAE; (**d**) Conjugated MMAE in plasma and tumor.

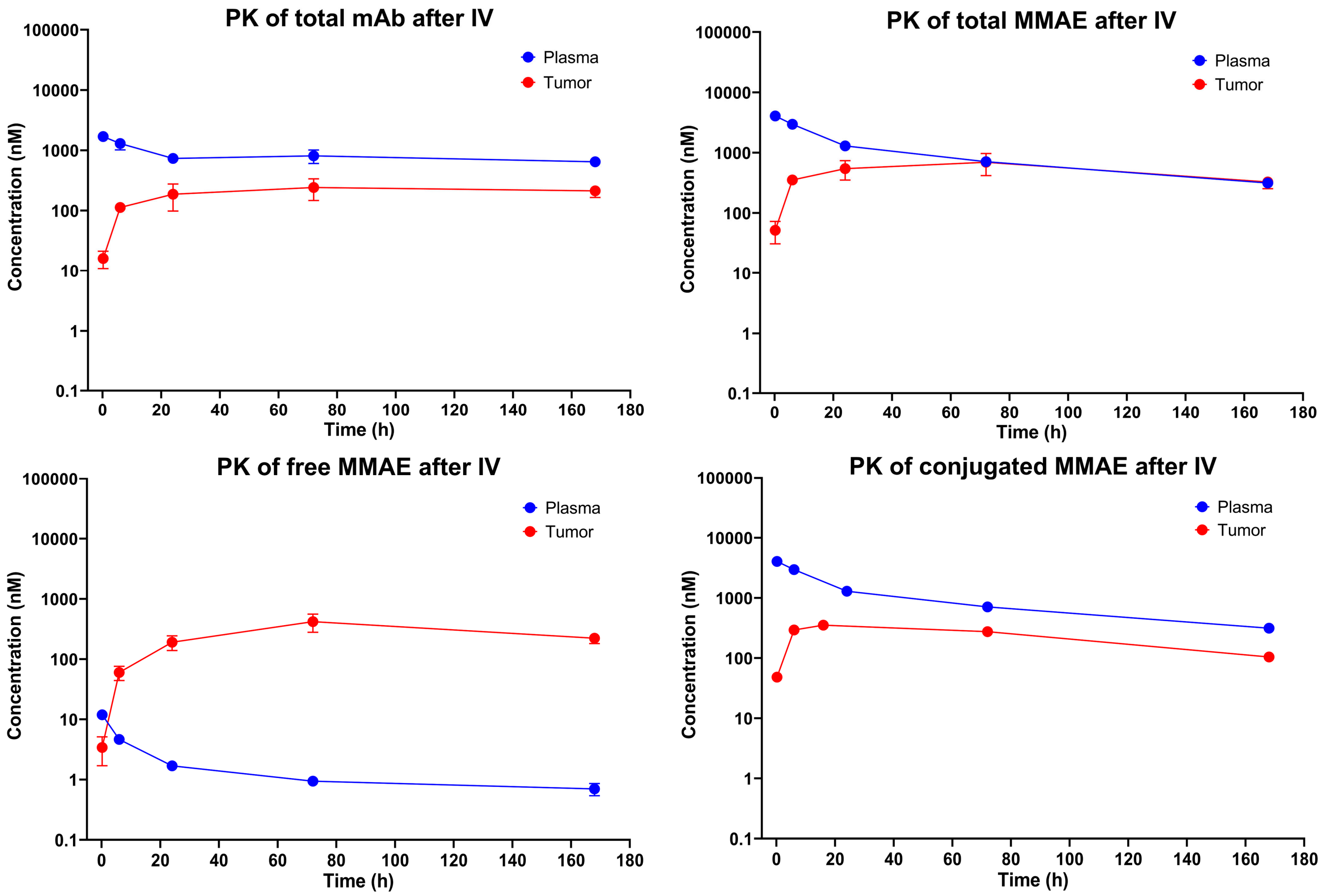

Figure S4. Observed plasma and tumor pharmacokinetics (PK) of ADC analytes in mice after intravenous (IV) administration of 10 mg/kg of T-vc-MMAE single dose. The figure displays the mean (SD) observed concentration of: (**a**) Total antibody; (**b**) Total MMAE; (**c**) Unconjugated MMAE; (**d**) Conjugated MMAE in plasma and tumor.

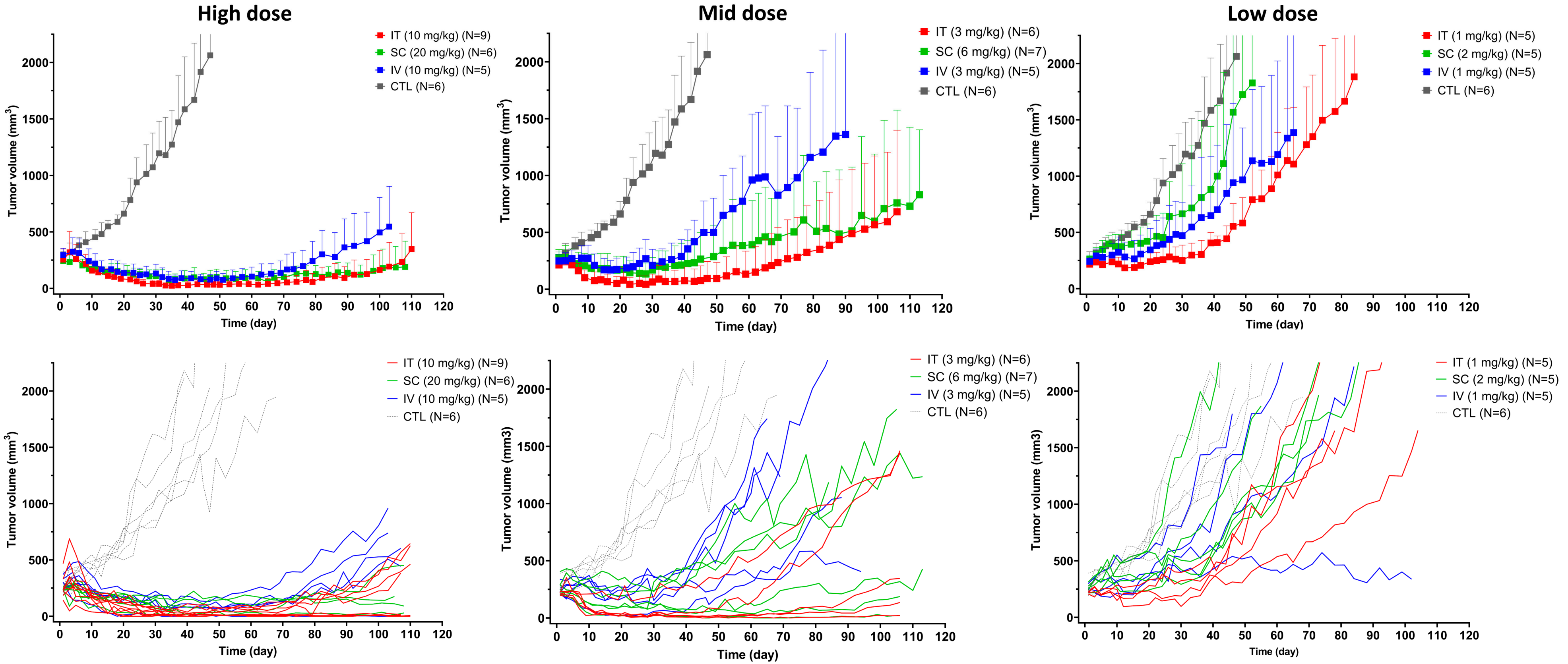

Figure S5. *In vivo* efficacy of T-vc-MMAE ADC after intravenous (IV), intratumoral (IT), and subcutaneous (SC) administration of high-, mid-, and low-doses of T-vc-MMAE. The figures show the mean (SD) tumor growth curves (upper) and individual tumor growth curves from each animal (lower) after treatment of high-, mid-, and low-dose of T-vc-MMAE single dose administered via IV, IT, and SC routes, along with the untreated group.

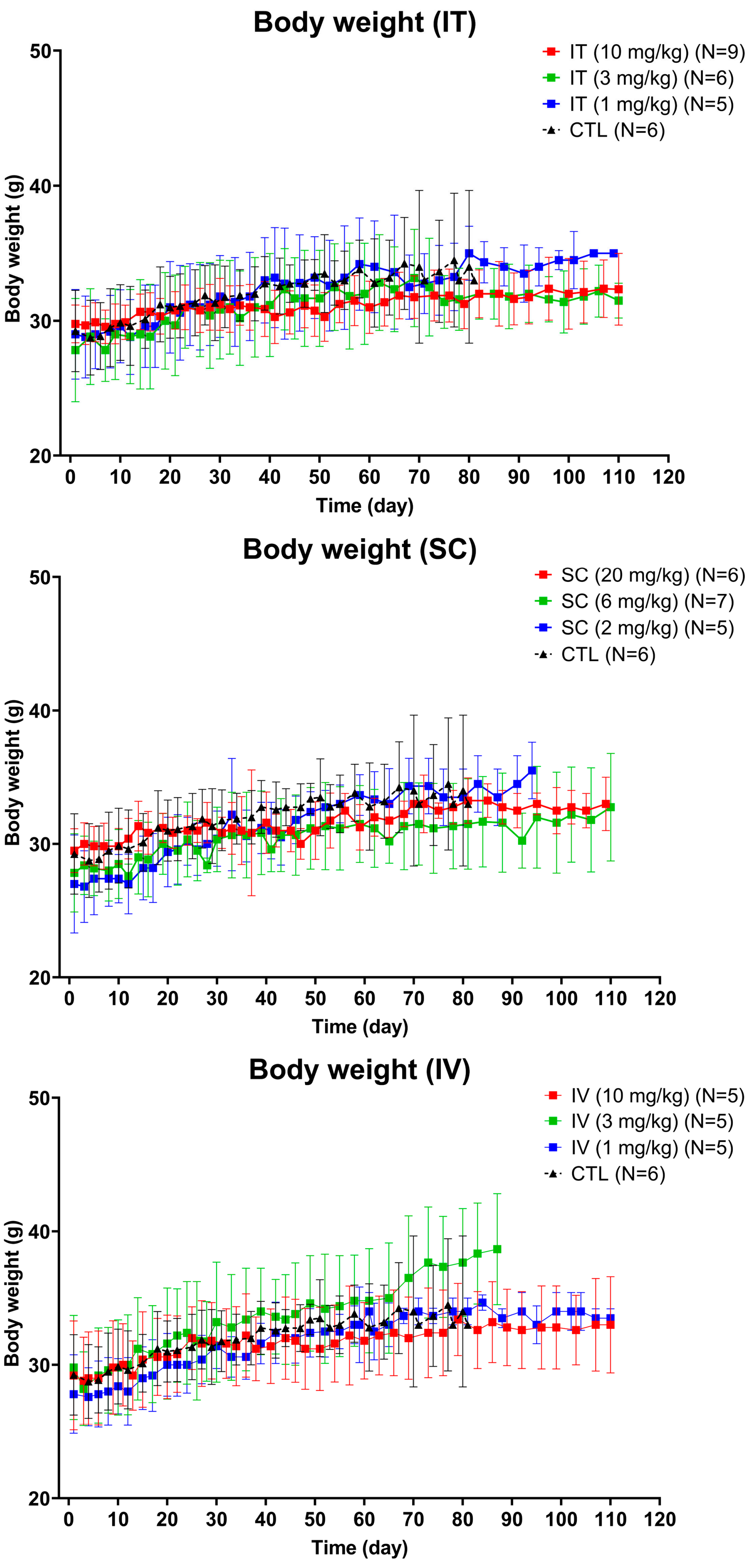

Figure S6. Body weight of mice included in the efficacy study. The figures display the mean (SD) body weight curves from mice receiving intratumoral (10, 3, 1 mg/kg), subcutaneous (20, 6, 2 mg/kg), and intravenous (10, 3, 1 mg/kg) administration of T-vc-MMAE single dose, along with the untreated group.

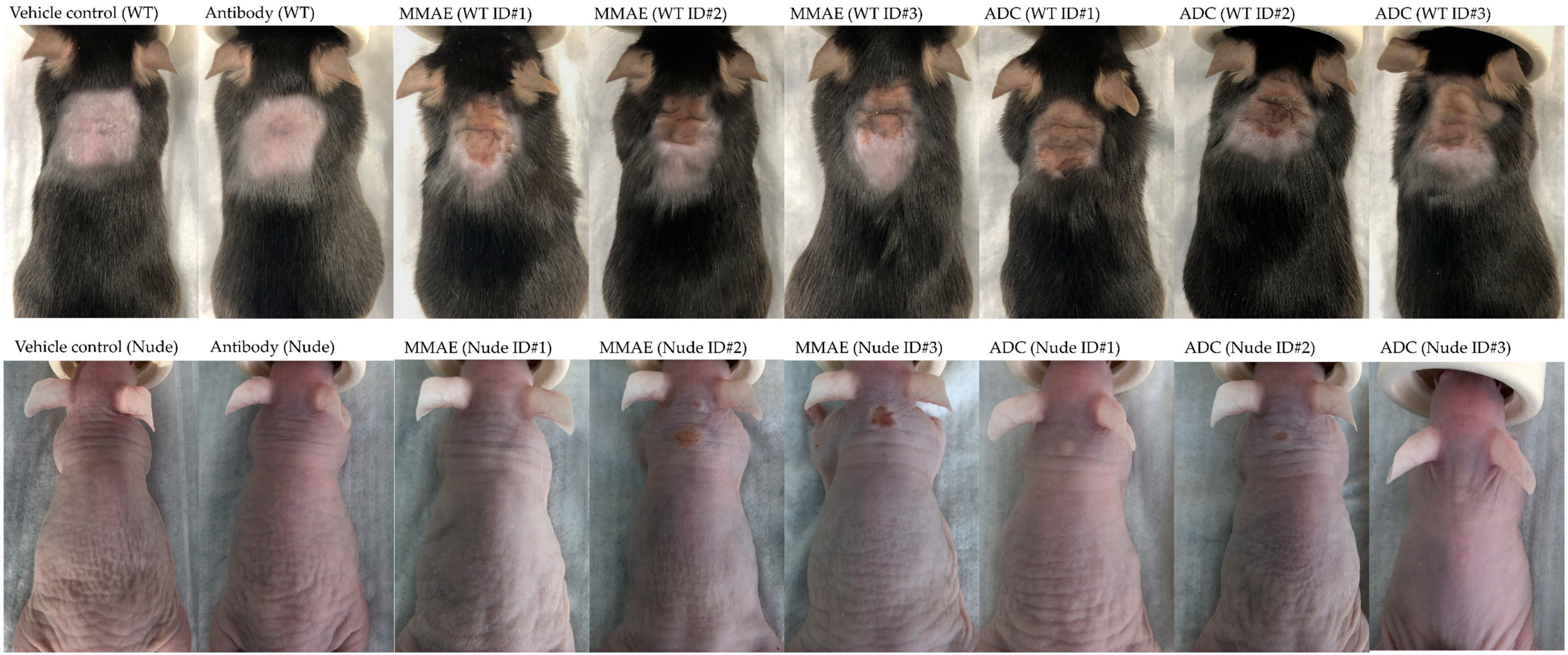

Figure S7. Skin observation at the injection site for WT (upper) and nude mice (lower) that received (from left to right): vehicle control subcutaneously, 30 mg/kg of trastuzumab single dose subcutaneously, 0.5 mg/kg of MMAE single dose subcutaneously (N = 3), and 30 mg/kg of T-vc-MMAE ADC single dose subcutaneously (N = 3).

Table S1. Summary of animal numbers and initial tumor volume of efficacy study for each administration route and each dosing level.

Sample size and Initial tumor volume				
Route	Dose (mg/kg)	N	Mean volume (mm^3)	Mean volume (mm^3)
IT	10	9	255 ± 94	255 ± 88
	3	6	248 ± 84	
	1	5	262 ± 99	
SC	20	6	246 ± 11	265 ± 45
	6	7	283 ± 66	
	2	5	261 ± 36	
IV	10	5	296 ± 60	262 ± 54
	3	5	249 ± 54	
	1	5	243 ± 38	
CTL	-	5	244 ± 75	246 ± 63
Total	-	58	258 ± 65	-
